# A GasPak-Based Ischemia Model for Studying ER Stress–Ischemia Interactions in Human Endothelial Cells

**DOI:** 10.3390/mps9020039

**Published:** 2026-03-04

**Authors:** Mathilde Hoareau, Grégorie Lebeau, Luce Muzi, Jérémy Fontaine, Pascale Krejbich-Trotot, Olivier Meilhac, Christine Robert-Da Silva, Wildriss Viranaicken

**Affiliations:** 1 Université de La Réunion, INSERM, UMR 1188 Diabète Athérothrombose Thérapies Réunion Océan Indien (DéTROI), 97410 Saint-Pierre, La Réunion, France; mathildehoareau22@gmail.com (M.H.); greg.lebeau@live.fr (G.L.); luce.muzi@univ-reunion.fr (L.M.); jeremytraill25@gmail.com (J.F.); olivier.meilhac@inserm.fr (O.M.); 2 Université de la Réunion, INSERM U1187, CNRS UMR 9192, IRD UMR 249, Unité Mixte Processus Infectieux en Milieu Insulaire Tropical, Plateforme Technologique CYROI, 94791 Sainte Clotilde, La Réunion, France; pascale.krejbich@univ-reunion.fr

**Keywords:** ischemia, endothelial cell, ER stress, apoptosis, oxygen deprivation

## Abstract

During ischemia, endothelial cell integrity is compromised, as a consequence, blood barrier homeostasis is disrupted. Therefore, the structural and functional preservation of endothelial cells is paramount when trying to improve outcomes after ischemic injury. Endoplasmic reticulum (ER) stress is increasingly recognized as a key player in ischemic injury through unfolded protein response (UPR) signalling, and its crosstalk with mitochondrial death pathways. This study provides a cost-effective and straightforward method to delve into the relationship between ER stress and ischemia in human microvascular endothelial cells-1 (HMEC-1). HMEC-1 was exposed to 8 h of oxygen–glucose deprivation (OGD) in glucose-free medium with rapidly induced hypoxia. Hypoxia, oxygen consumption, cell viability, apoptosis, and ER stress markers (BiP/GRP78, PERK, ATF6, IRE1/XBP1s, CHOP) were assessed by RT-qPCR and Western blot. Cell viability decreased by approximately 33% following OGD, while CHOP expression increased ~4-fold, indicating significant ER stress induction. The model enables quantification of metabolic stress (OCR), as well as evaluation of viability loss, membrane integrity, apoptotic commitment, and discrimination between ER stress resolution versus maladaptation. Overall, GasPak EZ Pouch Systems provide a reproducible and practical in vitro platform to study ischemic injury down to the mechanistic details of ER-mitochondria signalling. They give the opportunity to evaluate therapeutic approaches that target ER homeostasis to limit apoptosis and/or recovery of metabolic function after ischemia. This method could allow rapid screening of ER stress-modulating interventions aimed at preserving endothelial barrier function, in various ischemic contexts.

## 1. Introduction

Ischemia, which is defined as an insufficient supply of oxygen and nutrients due to impaired blood flow, constitutes a central pathological mechanism underlying a wide scope of both acute and chronic diseases such as myocardial infarction, peripheral artery disease, cerebral ischemia, and organ failure. Pathologies stemming from hypoxia represent a considerable burden and are prevalent amongst individuals exposed to well-known risk factors such as smoking, diabetes, hypertension and hyperlipidemia. The deprivation of glucose and oxygen during ischemia imposes profound metabolic stress on tissues. Such stress can be exacerbated upon reperfusion and lead to oxidative stress, inflammation, as well as mitochondrial dysfunction. Endothelial cells form the inner lining of the blood vessels and play a pivotal role in maintaining vascular homeostasis through regulation of barrier function, vascular tone, and metabolic exchange. Endothelial cells can also drive key adaptive responses, such as angiogenesis and inflammation, and engage cell trophic signaling pathways. However, after an ischemic event, endothelial cells become dysfunctional, which leads to increased vascular permeability, altered hemodynamics, thrombovascular complications, and tissue injury. Therefore, preserving the structural and functional integrity of endothelial cells emerges as a key therapeutic target to improve outcomes in ischemic conditions across different organs. Since endothelial cells represent a primary target of ischemia-induced injury, we selected an immortalized endothelial cell line model to investigate the molecular mechanisms driving endothelial dysfunction. Our objective was to study the interplay between ischemia and ER stress using an oxygen–glucose deprivation protocol (OGD) in human microvascular endothelial cells-1 (HMEC-1), and to characterize the subsequent cell death pathways activated.

Hypoxia, resulting from insufficient oxygen availability, is a hallmark of ischemic injury and imposes profound metabolic stress on cells. Hypoxia can lead to cell death through energy depletion and excessive reactive oxygen species production. Both conditions disturb endoplasmic reticulum (ER) homeostasis [1], and as a result, cells activate adaptive responses that reduce energy expenditure and optimize both nutrient usage and availability, ultimately aiming at promoting survival. The unfolded protein response (UPR) is triggered by an accumulation of misfolded or otherwise faulty proteins signalled by the chaperone BiP/GRP78 within the ER lumen. The UPR aims at restoring ER homeostasis through three main signaling branches, depending on PERK, ATF6 and IRE1, respectively. While initially cytoprotective, unresolved or prolonged UPR activation shifts cells towards death pathways. This shift is directed by a crosstalk with mitochondria via B-cell lymphoma protein 2 (Bcl-2) family proteins, and can trigger either autophagy or apoptotic death pathways. Consequently, the UPR plays a crucial role in determining cell fate in ischemic stress through mitochondrial signaling [2]. This ER–mitochondrial crosstalk can be explored by combining the assessment of UPR activation with the evaluation of downstream markers of mitochondrial dysfunction (metabolic shift) and/or mitochondrial-dependent death pathways (BAX activation).

C/EBP homologous protein (CHOP), also known as GADD153 (growth arrest and DNA damage-inducible gene 153), plays a pivotal role in switching the UPR from an adaptive to a pro-apoptotic program [3].

Oxygen–glucose deprivation (OGD) is the most widely used in vitro method to reproduce ischemic injury [4]. In practice, OGD consists of replacing the standard culture medium with a glucose-free medium under hypoxic conditions. Several approaches can be used to induce hypoxia in cell culture, ranging from controlled-environment chambers to chemical mimetics (Table 1). The oxygen scavenging method directly depletes oxygen, whereas chemical mimetics act indirectly by inhibiting prolyl hydroxylases that target HIF-1α for degradation. The main limitations inherent to this method are the difficulty in maintaining stable oxygen levels over extended periods as well as the potential change of pH and nutrient levels in the cell culture medium. These minor drawbacks were deemed acceptable considering the speed and efficiency of the method. Furthermore, unlike hypoxia chambers, which require costly equipment, the oxygen scavenging system remains inexpensive. Oxygen scavenging also rapidly induces hypoxia, and can be performed in standard labs with cell culture facilities in small-scale experiments. For our OGD experiment, we used an oxygen scavenging system named GasPak. This method allows analysis of energy-deprivation-mediated biochemical and molecular mechanisms, and enables direct analysis of key regulatory pathways involved in necrosis and apoptosis. The OGD model also provides a robust platform to evaluate potential therapeutic interventions to prevent or attenuate ischemia-induced cell damage.

## 2. Materials and Methods

### 2.1. Cell Culture

Human microvascular endothelial cells (HMEC-1) (ATCC, CRL- 3243™) were cultured in Dulbecco’s Modified Eagle’s Medium (DMEM) containing 1 g·L^−1^ glucose (PAN Biotech, Aidenbach, Germany). The medium was supplemented with 10% fetal calf serum, 2 mM L-glutamine, 100 U·mL^−1^ penicillin, 0.1 mg·mL^−1^ streptomycin, and 0.5 μg·mL^−1^ amphotericin B (PAN Biotech, Aidenbach, Germany). The medium was further supplemented with hydrocortisone (1 µg·mL^−1^) and human epidermal growth factor (10 ng·mL^−1^) (Sigma-Aldrich, Humeau, La Chapelle-Sur-Erdre, France). Cells were maintained in normoxia condition at 37 °C in a humidified 5% CO_2_ atmosphere. The day before experiments HMEC-1 cells were seeded at a density of 1000 cells/mm^2^. Cells were incubated overnight to allow adhesion and to reach subconfluence at the time of the experiment.

### 2.2. Oxygen–Glucose Deprivation

To initiate oxygen–glucose deprivation (OGD), the culture medium was removed, and the cells were washed with phosphate-buffered saline (1X PBS) and incubated in glucose-free DMEM containing antibiotics/antimycotics. Instead of using a conventional hypoxia chamber, an anaerobic atmosphere was generated by sealing confluent plates in a GasPak EZ Pouch System (BD, Le Pont de Claix, France). The plates were subsequently incubated at 37 °C for 8 h. The induction of hypoxia was confirmed using Anaer Indicator from BioMérieux (Marcy-l’étoile, France).

### 2.3. EF5 Staining

To detect cell hypoxia, EF5 (100 µM; CAS 152721-37-4, Merck, Humeau, La Chapelle-Sur-Erdre, France), a pentafluorinated derivative of etanidazole metabolically reduced by oxygen-inhibitable nitroreductase, was added during OGD. After treatment, HMEC-1 cells cultivated on coverslips were fixed with 4% PFA in 1X PBS, for 10 min, then rinsed three times in 1X PBS. Blocking was conducted for 1 h at room temperature with 1% BSA in PBT (1X PBS + 0.1% Triton). Cells were incubated overnight at 4 °C with a mouse monoclonal anti-EF5 antibody (clone ELK3-51, Alexa Fluor 488-conjugated; Merck EF5-30A4; 1:1000 in 1% BSA/PBT). DNA was counterstained with 4′, 6-diamidino-2-phenylindole dihydrochloride (DAPI) (1:2000). Cells on coverslips were rinsed with PBST for 5 min, mounted with an aqueous mounting medium, and imaged by fluorescence microscopy using filters appropriate for DAPI and Alexa Fluor 488.

### 2.4. Immunofluorescence Assay

Confluent HMEC-1 cells grown on coverslips were fixed in 4% PFA in 1X PBS, washed three times in 1X PBS. Cells were permeabilized with 0.3% Triton X-100 and incubated for 1 h at room temperature with rabbit anti-BAX (Cell Signaling Technology, Ozyme, Saint-Cyr-l’École, France; 1:1000 in PBS with 1% BSA) and mouse anti-CHOP (Clone 9C8, Thermofisher, Les Ulis, France; 1:1000 in PBS with 1% BSA). After three washes in 1X PBS, samples were incubated with donkey anti-mouse Alexa Fluor 594, and anti-rabbit Alexa Fluor 488 (Invitrogen, Thermofisher, Les Ulis, France; 1:1000 in 1X PBS containing 1% BSA). Cells were washed three times in 1X PBS, mounted with VectaShield containing DAPI (Cedarlane, Burlington, ON, Canada), and stored at 4 °C. Fluorescence images were captured using a Nikon Eclipse E2000-U microscope equipped with a Hamamatsu ORCA2 ER camera (Nikon, Tokyo, Japan) and processed with AR version of NIS-Element software.

### 2.5. Cell Viability Assays

#### 2.5.1. MTT Assay

Cell viability was assessed with an MTT assay (3-(4, 5-dimethylthiazol-2-yl)-2, 5-diphenyltetrazolium bromide). After 8 h OGD, 20 μL of 5 mg·mL^−1^ MTT reagent (Sigma-Aldrich, Humeau, La Chapelle-Sur-Erdre, France) was added to each well. Cells were incubated at 37 °C for 2 h. After incubation, the medium was gently removed, and formazan crystals were dissolved in 100 μL DMSO. Absorbance was measured at 570 nm using a FLUOstar^®^ Omega (BMG LABTECH, Offenburg, Germany).

#### 2.5.2. Neutral Red Assay

Cell viability was assessed by neutral red uptake. After 8 h OGD, the culture medium was removed, and 100 µL of medium (DMEM with glucose 1 g·L^−1^) supplemented with 40 µg·L^−1^ of neutral red was added to each well. Cells were incubated at 37 °C for 2 h. After incubation, the medium was discarded, and the cells were washed with 1X PBS. Subsequently, 150 µL of neutral red destain solution (50% ethanol, 49% H_2_O, 1% glacial acetic acid) was added to each well. Absorbance was measured at 540 nm using FLUOstar^®^ Omega microplate reader (BMG LABTECH, Offenburg, Germany).

#### 2.5.3. Annexin V/PI Flow Cytometry

To monitor apoptotic and necrotic cell subpopulations, HMEC-1 cells were stained with Annexin V and Propidium iodide (PI) using the Annexin V-FITC Apoptosis Detection Kit (Ab14085, Abcam, Cambridge, UK) according to the manufacturer’s instructions. Following 8 h OGD, cells were washed with 1X PBS, detached with trypsin/EDTA, collected, and pelleted at 800× *g* for 5 min at 4 °C. Pellets were washed with 1X PBS to remove residual EDTA and resuspended in 500 μL of 1X Annexin V binding buffer, then adjusted to 5 × 10^5^ cells per tube. Annexin V (5 µL) and Propidium iodide (5 μL) were added, gently mixed, and incubated for 5 min at room temperature in the dark. Cells were analyzed by flow cytometry using a CytoFLEX (Beckman Coulter, Villepinte, France). The gating strategy consisted of sequential exclusion of debris, doublets, and non-viable events (FSC/SSC), followed by Annexin V and PI based discrimination of viable, early apoptotic, and late apoptotic/necrotic cells. A representative example is shown in Supplementary Figure S2.

### 2.6. ATP Content

Intracellular ATP levels in HMEC-1 cells were measured using the Viral ToxGlo^TM^ assay kit (Promega, Madison, WI, USA) according to the manufacturer’s instructions. Briefly, ATP Detection Reagent provided by the manufacturer was added to each well at a 1:1 ratio with the culture medium, mixed briefly, and incubated for 10 min at room temperature. Luminescence was quantified using a FLUOstar^®^ Omega microplate reader (BMG LABTECH, Offenburg, Germany).

### 2.7. Oxygen Consumption Rate Assessment

HMEC-1 cells were cultured in 25 cm^2^ flasks under either normoxic or hypoxic conditions for 8 h, rinsed with 1X PBS, detached using trypsin/EDTA, and centrifuged at 800× *g* for 5 min at room temperature. Pelleted cells were resuspended in DMEM containing 1 g·L^−1^ glucose and adjusted to 2 × 10^6^ cells per chamber. Basal oxygen consumption rates (OCR) were continuously monitored at 37 °C in intact cells using a Clark-type oxygen electrode (Oroboros Oxygraph-2k (O_2_k), Oroboros Instruments, Innsbruck, Austria). For sensor calibration and background oxygen consumption measurements, a medium without biological samples was introduced into the O_2_k-chamber. OCR was assessed for each experimental condition with sequential additions of specific inhibitors: F1F0-ATP synthase inhibitor oligomycin (1 μM), protonophoric uncoupler dinitrophenol (100 μM), and a combination of mitochondrial complex III inhibitor myxothiazol (1 µM) and mitochondrial complex I inhibitor rotenone (1 μM). Data were expressed as picomoles O_2_ consumed per second and per millions of cells.

### 2.8. Western Blot

Cells were collected in TEN buffer (0.1 M Tris-Cl pH 9.0, 0.01 M EDTA, and 1 M NaCl) and lysed by sonication on ice for 1 min at 40% amplitude in 0.5-cycle pulse mode using an Omni Ruptor 4000 (Omni International, Dutscher, Issy-les-Moulineaux, France). Total cell extract protein content was quantified by BCA assay according to the manufacturer’s instructions (Sigma-Aldrich, Humeau, La Chapelle-sur-Erdre, France). Equal protein amounts were mixed with Laemmli buffer containing 10 mM DTT and heated at 95 °C for 5 min. Protein samples (15 µg per lane) were loaded in duplicate and separated on a 12% SDS-PAGE gel at a constant voltage of 150 V for 1 h, and transferred to a 0.45 µm nitrocellulose membrane (Protan, Amersham, GE, Buc, France) using a semi-dry system at 60 mA per gel for 2 h. Following transfer, the membrane was cut in half. Each half was blocked in 5% skimmed powder milk in PBS-T (1X PBS, 0.1% Tween-20), then one half was incubated overnight at 4 °C with primary antibodies: mouse monoclonal anti-CHOP, (9C8, ThermoFisher; 1:1000 in PBS-T); in parallel, the other half was incubated with mouse monoclonal anti-alpha Tubulin (DM1A, Sigma-Aldrich; 1:1000 in PBS-T). After three washes in PBS-T, half-membranes were incubated for 1 h with goat anti-mouse secondary antibody (immunoglobulin-horseradish peroxidase conjugate, Jackson ImmunoResearch; 1:2000 in PBS-T with 1% skimmed powder milk). Protein bands were visualized using enhanced chemiluminescence (ECL) prime detection reagents (GE, Buc, France) and an Amersham Imager 680 (GE, Buc, France).

### 2.9. Reverse Transcription-Quantitative Polymerase Chain Reaction (RT-qPCR)

Total RNA was extracted from cell lysates using the RNeasy Plus Mini Kit (cat. 74136, Qiagen, Hilden, Germany). Subsequently, cDNA was synthesized using random primers (Invitrogen, ref. 58875, Thermofisher, Les Ulis, France) and M-MLV reverse transcriptase enzyme (ref. M1708, Promega, Madison, WI, USA) at 42 °C for 60 min. Quantitative polymerase chain reaction (qPCR) was performed on the cDNA using a CFX96 Connect™ Real Time Detection System (Bio-Rad, Hercules, CA, USA). Amplification was performed with SYBRGreen (Applied Biosystems) and specific human primers targeting the following genes: CHOP (F: 5′-ATGGCAGCTGAGTCATTGCC-3′, R: 5′-CATTTTCATCTGAAGACAGG-3′), ATF4 (F: 5′-TGACCTGGAAACCATGCCAG-3′, R: 5′-AATGATCTGGAGTGGAGGAC-3′), XBP1s (F: 5′-CTGAGTCCGCAGCAGGTG-3′, R: 5′-ATCCATGGGGAGATGTTCTGG-3′), TRB3 (F: 5′-TGGTACCCAGCTCCTCTACG-3′, R: 5′-GACAAAGCGACACAGCTTGA-3′), and RNA polymerase II (F: 5′-GAGAGCGTTGAGTTCCAGAACC-3′, R: 5′-TGGATGTGTGCGTTGCTCAGCA-3′). The threshold cycle (Ct) was determined for each sample amplification reaction in the exponential phase using Bio-Rad CFX Manager 3.1 (Bio-Rad, Hercules, CA, USA). The data were processed using the 2^−ΔΔCt^ method, with RNA polymerase II serving as the housekeeping gene.

### 2.10. Statistical Analyses

Statistical analyses were performed in Graph-Pad Prism software version 9 using *t*-test correction or two-way ANOVA as appropriate. Values of *p* < 0.05 were considered statistically significant. The degrees of significance are indicated in the figures captions as follows: * *p* < 0.0332; ** *p* < 0.0021; *** *p* < 0.0002; **** *p* < 0.0001.

## 3. Results

### 3.1. GasPak OGD Induces Hypoxia in HMEC-1 Cells

#### 3.1.1. Establishing the *In Vitro* OGD Model

In comparison with existing approaches for inducing hypoxia in vitro (Table 1), we chose to implement an oxygen-scavenging strategy using the GasPak EZ system. While methods such as hypoxia chambers, controlled-atmosphere incubators, enzymatic oxygen depletion, or chemical hypoxia mimetics each offer specific advantages, they often require costly equipment, complex setup, or carry limitations in physiological relevance. GasPak, routinely used for the cultivation of anaerobic bacteria, provides a simple, inexpensive, and equipment-free alternative capable of rapidly reducing oxygen levels within sealed pouches. This approach therefore represents an accessible and reproducible solution for laboratories seeking to model ischemic conditions without specialized infrastructures, while maintaining a tightly controlled hypoxic environment.

**Figure 1:**
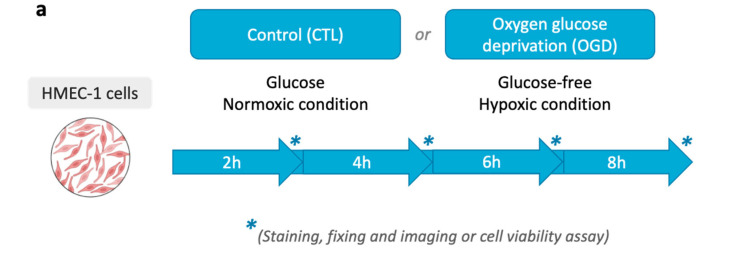

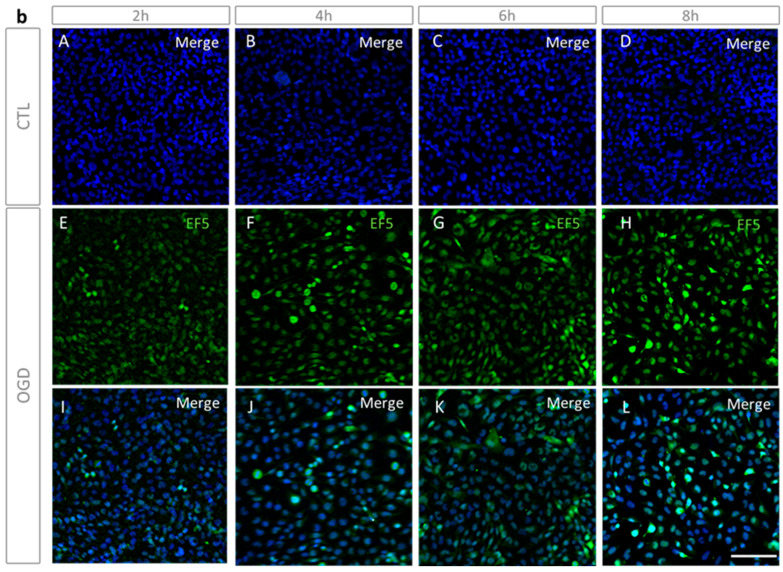
Hypoxia detection via EF5 following OGD. (**a**) Experimental protocol. For OGD, cells were incubated in glucose-free DMEM and placed at 37 °C, in a GasPak (EZ Pouch Systems) for 2, 4, 6, or 8 h. Hypoxia was then assessed by EF5 staining. (**b**) EF5 immunofluorescence after OGD. HMEC-1 was subjected to 2, 4, 6, or 8 h of OGD or maintained under normoxic conditions for the same duration (CTL); EF5 (green) and nuclei (DAPI, blue) are shown with merged images indicated (EF5 + DAPI). Scale bar: 30 µm (**A**–**L**).

We first tested oxygen deprivation with Anaer Indicator as indicated in materials and methods in the GasPak bag. The absence of oxygen is achieved after 30 min, based on the indicator strip. We next verified, as a preliminary step, that the GasPak setup can induce hypoxia in HMEC-1 cells. To do so, cells were incubated in glucose-free DMEM, and placed inside a GasPak anaerobe pouch at 37 °C for 2, 4, 6, or 8 h (Figure 1a). Then EF5, a nitroreductase-dependent hypoxia probe, was used to ascertain hypoxia (Figure 1b).

Alexa Fluor 488-conjugated anti-EF5 antibody staining revealed a time-dependent increase in fluorescence across 2, 4, 6, and 8 h of OGD (Figure 1b). First, based on the progressive increase in EF5 staining across the OGD time course, we focused subsequent analyses on the 8 h condition, which consistently produced the most pronounced hypoxic signal. Importantly, this longer OGD duration also provides a more robust window for downstream biological responses to emerge. Hypoxia-related transcriptional and protein-expression, such as HIF activation, metabolic enzyme, ER stress responses, and pro-inflammatory signalling, typically require several hours to reach detectable levels [13,14]. Thus, the 8 h time point maximizes the likelihood of capturing a broad spectrum of molecular events, which are essential for mechanistic interpretation. It would improve discrimination between experimental conditions, such as pharmacological interventions or preconditioning treatments in future use of this protocol, allowing for testing of cytoprotective compounds on the molecular events defined above. Finally, with these results, and in alignment with previous reports on endothelial cell viability by Chen et al. [15], an 8 h OGD exposure was selected for further experiments to characterize cell responses to hypoxia with GasPak.

#### 3.1.2. OGD Alter Mitochondria Function and Metabolic Plasticity in HMEC-1 Cells

Mitochondria, the primary consumer of oxygen, is severely affected by oxygen deprivation. Hypoxia-driven regulation has recently emerged as crucial in mitochondrial dysfunction [16]. In this context, we analyzed whether OGD induces mitochondrial respiration dysfunction after recovery of live cells in complete media.

**Figure 2:**
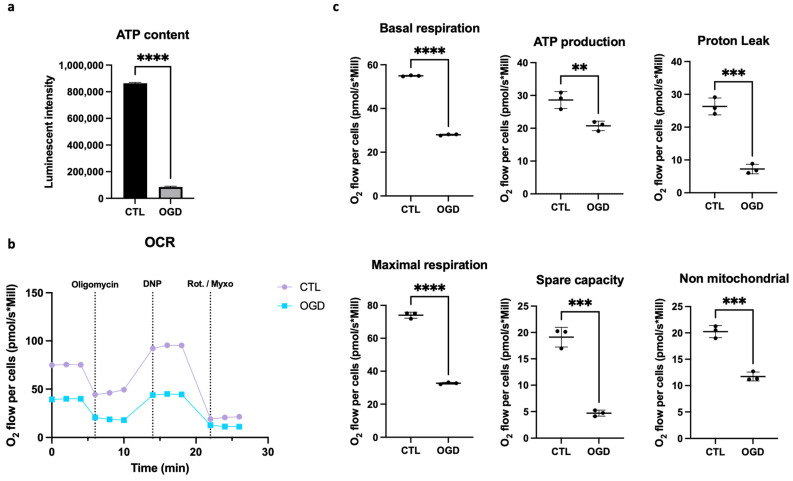
Mitochondrial respiration dysfunction in HMEC-1 during OGD. (**a**) Assessment of ATP content through Viral ToxGlo^TM^ assay. In HMEC-1 cells, OGD significantly reduces ATP relative to control. (**b**,**c**) Oxygen consumption rate (pmol/s·Mill) in HMEC-1 cells under control or OGD conditions. Sequential injections of: oligomycin (1 µM, ATP synthase inhibitor), 2,4-dinitrophenol (DNP, 100 µM, protonophoric uncoupler to elicit maximal respiration), and rotenone (1 µM) with Myxothiazol (1 µM, complex I and III inhibitors to determine non-mitochondrial respiration) were realized. OGD reduces basal O_2_ consumption flux, ATP production, proton leak, maximal respiration, spare capacities, and non-mitochondrial respiration. Error bars represent standard errors of three independent experiments. ** *p* < 0.0021; *** *p* < 0.0002; **** *p* < 0.0001; unpaired *t*-test.

As shown in Figure 2a, oxygen–glucose deprivation caused a significant decrease in ATP. Since ATP production depends on the availability of glucose and oxygen, and functions primarily through glycolysis and oxidative phosphorylation, we therefore evaluated mitochondrial respiration by comparing oxygen consumption rates after control or OGD conditions when the cells are placed in normal condition for oxymeter analysis (Figure 2b). In line with the ATP assay, we showed that OGD lowered basal oxygen consumption, which indicates an overall reduction in cellular respiration under OGD conditions. Moreover, OGD lowered ATP production, which aligns with decreased oxygen consumption, as oxygen is required for ATP production by the mitochondrial electron transport chain. In addition, proton leak was diminished under OGD conditions, which suggests that fewer protons cross the mitochondrial membrane without contributing to ATP synthesis. This may point towards impaired mitochondrial function. Maximal respiration (the maximum respiratory capacity in the presence of an uncoupler) was also reduced, which indicates the cells’ inability to increase their oxygen consumption adequately when energy demands rise. Besides, spare respiratory capacity (the difference between maximal and basal respiration) was reduced, which reflects a diminished metabolic flexibility after stress. Finally, non-mitochondrial respiration was also lowered, indicating a reduction in mitochondria-independent oxygen consumption. Taken together, these data suggest that OGD promotes impairment of the mitochondria functionality of HMEC-1.

### 3.2. OGD Causes Cell Death in HMEC-1 Cells

During ischemia, cells undergo severe metabolic stress due to limited oxygen and glucose [17]. In line with the above results for metabolic impairment, we therefore assessed cell viability during OGD in HMEC-1 cells.

**Figure 3:**
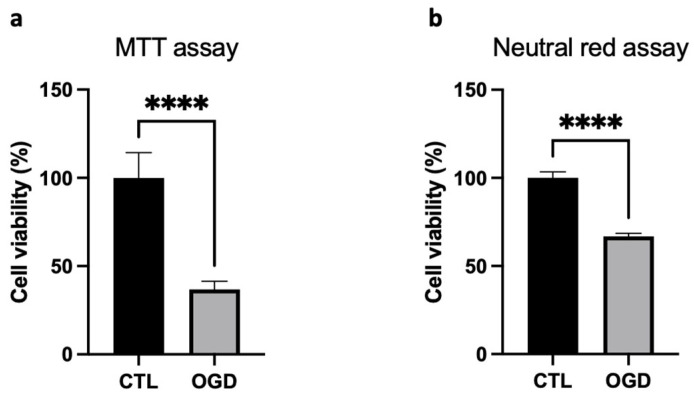
Impact of OGD on the viability of HMEC-1 cells. OGD reduces cell viability in HMEC-1 as measured by MTT (**a**) and neutral red uptake assays (**b**). Error bars represent standard errors of three independent experiments; unpaired *t*-test. **** *p* < 0.0001; *t*-test.

Cell viability was evaluated through complementary assays to provide an integrated assessment. First, the MTT assay quantified cellular reductase activity, thereby providing a reading of mitochondrial function. Since the MTT assay may bias the estimation of cell viability under our conditions due to metabolic changes, as described above, we performed an additional test, the Neutral Red Uptake (NRU) assay. The NRU assay offered an evaluation of lysosomal integrity, independently of mitochondrial function. The discrepancy observed between MTT and NRU results likely reflects these differences in the assessed cellular processes. After OGD, HMEC-1 cells exhibited approximately 37% viability in the MTT assay (Figure 3a) and about 67% viability in the Neutral Red uptake assay (Figure 3b). As anticipated, the MTT assay overestimates cell mortality; nevertheless, cell death is clearly observed with the NRU assay.

### 3.3. OGD Induces Apoptosis in HMEC-1 Cells

**Figure 4:**
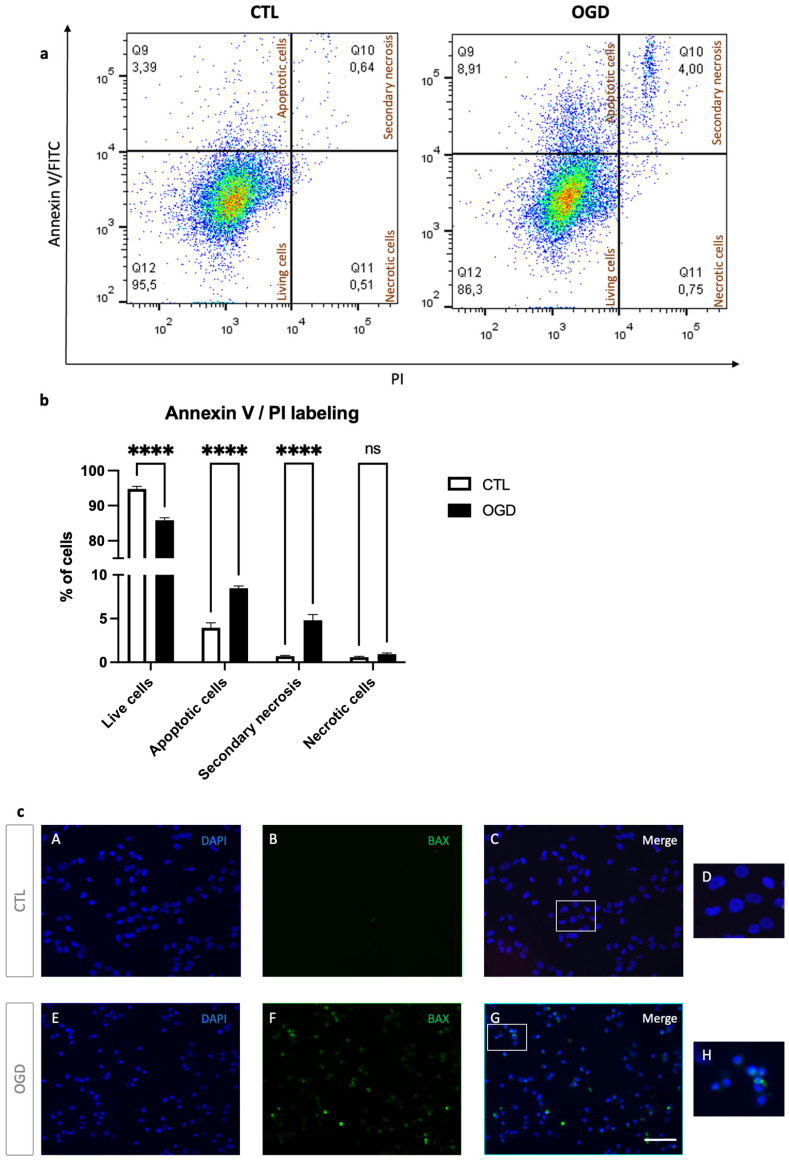
OGD induces apoptosis in HMEC-1 cells. (**a**) After 8 h of OGD, HMEC-1 cells were stained with Annexin V-FITC and Propidium iodide and analyzed by flow cytometry. Quadrant analysis of Annexin V-FITC/PI flow cytometry of HMEC-1. Percentages of each population are indicated within the corresponding quadrants: Q12, viable cells (Annexin V-, PI-), Q9, early apoptotic cells (Annexin V+, PI-), Q10, secondary necrotic cells (Annexin V+, PI+), and Q11, necrotic cells (Annexin V-, PI+). (**b**) Quantitative analysis of Annexin V-FITC/PI populations. In HMEC-1 cells, OGD increased early apoptotic (Annexin V+, PI-) and late apoptotic/secondary necrotic cells (Annexin V+, PI+), consistent with a decrease in live cells (Annexin V-, PI-). PI-only necrotic events (Annexin V-, PI+) were unchanged. Error bars represent standard errors of three independent experiments. ns = not significant; **** *p* < 0.0001; two-way ANOVA followed by Šídák’s multiple comparisons test (**c**) OGD in HMEC-1 cells increases punctuate BAX staining. Immunofluorescence detection for BAX (green) after 8 h OGD with 51.2% of positive cells and under control conditions (CTL) with no detection for punctuate. DNA was counterstained with DAPI (blue). CTL (**A**–**C**) and OGD (**E**–**G**). Panels (**D**,**H**) show magnified views of the areas indicated by the white rectangles in (**C**) and (**G**), respectively. Scale bar: 30 µm (**A**–**C**,**E**–**G**).

To better characterize cell death in our model, we next assessed apoptosis and necrosis by Annexin V/PI staining. For specific evaluation of mitochondrial-dependent cell death pathways, BAX immunolocalization were evaluated (Figure 4).

As shown in Figure 4a,b, OGD increased early apoptotic (Annexin V+, PI-) and late apoptotic/secondary necrotic cells (Annexin V+, PI+), with a corresponding decrease in live cells (Annexin V-, PI-). However, no significant difference was observed in the number of PI-only necrotic events (Annexin V-, PI+). In line with these data, BAX immunofluorescence showed clustered BAX consistent with mitochondrial relocalisation in HMEC-1 cells following OGD (Figure 4c). Together, these findings indicate that OGD induces apoptotic cell death with progression to secondary necrosis in HMEC-1 cells.

### 3.4. OGD Induces ER Stress and UPR Signaling in HMEC-1 Cells

Since prolonged ER stress-related perturbations are known to enhance UPR-mediated cytotoxicity and apoptosis [2], we focused on ER stress regulation, with particular attention on CHOP, a key transcription factor driving ER-stress-induced apoptosis [3].

**Figure 5:**
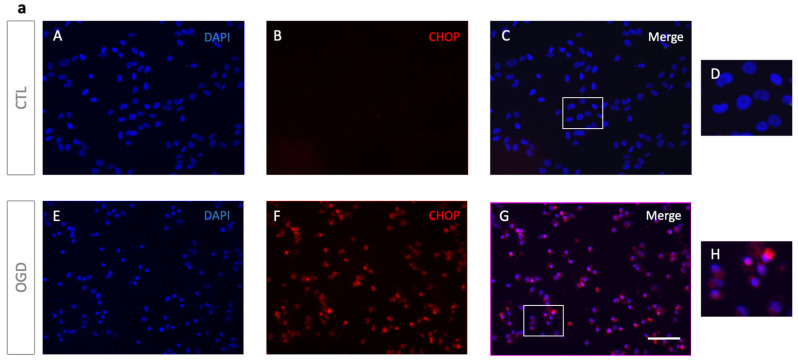

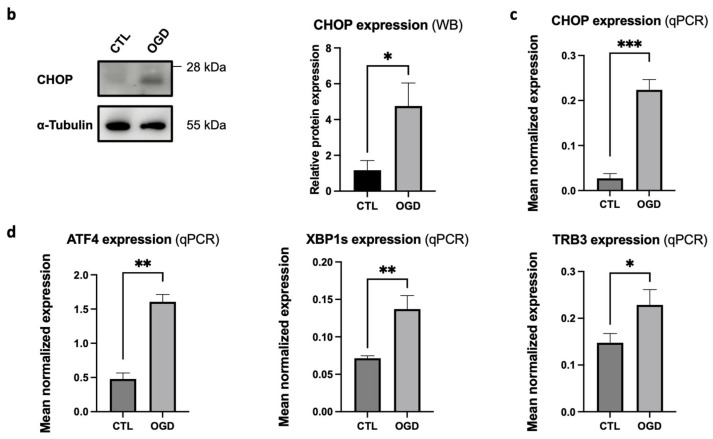
OGD induces ER stress and UPR activation in HMEC-1 cells. (**a**) OGD cells trigger CHOP induction in HMEC-1. CHOP signal is elevated after OGD with 83.87% of positive cells (**F**) but undetectable under normoxic conditions (**B**) in HMEC-1 cells. Immunofluorescence analysis shows CHOP (red) after 8 h of OGD or normoxic condition (CTL) in HMEC-1 cells. Nuclei were stained with DAPI (blue). Panels (**D**,**H**) show magnified views of the areas indicated by the white rectangles in (**C**) and (**G**), respectively. Scale bar: 30 µm (**A**–**C**,**E**–**G**). (**b**) Lysates from OGD-treated and control HMEC-1 cells were analysed by Western blot for CHOP. α-Tubulin was used as a loading control. Detection was performed using mouse anti-CHOP and mouse anti-α-tubulin antibodies. Blots shown are representative of three independent experiments. (**c**) Up-regulation of CHOP under OGD conditions. CHOP mRNA was quantified by RT-qPCR following OGD compared to cells in normoxic conditions (CTL). Results are mean normalized expression using RNA polymerase II (pol II) as a housekeeping gene. Error bars represent standard errors of four independent experiments. * *p* < 0.0332; ** *p* < 0.0021; *** *p* < 0.0002; *t*-test. (**d**) Up-regulation of ATF4, XBP1s, and TRB3 under OGD conditions. ATF4, XBP1s, and TRB3 transcripts were measured by RT-qPCR following OGD compared to cells under normoxic conditions (CTL). Values are mean-normalized expressions normalized to pol II. Error bars represent standard errors of three independent experiments. * *p* < 0.0332; ** *p* < 0.0021; *** *p* < 0.0002; *t*-test.

Immunofluorescence analysis showed a nuclear localization of CHOP in HMEC-1 cells after OGD, suggesting its involvement in an UPR-driven apoptosis induction. In contrast, normoxic controls exhibited minimal staining for CHOP (Figure 5a). Western blot (WB) analyses confirmed that CHOP levels increased after OGD when compared to normoxic conditions (Figure 5b). This was characterized both at the protein and transcript levels (Figure 5c). To further characterize ER stress following OGD, we assessed UPR-related genes downstream (TRB3) and upstream (ATF4, XBP1s) of CHOP (Figure 5d). As XBP1 splicing is uniquely mediated by IRE1 endoribonuclease activity, the quantification of XBP1s using splice-specific primers represents a specific molecular readout of IRE1 pathway activation, independent of total XBP1 transcriptional regulation.

**Figure 6:**
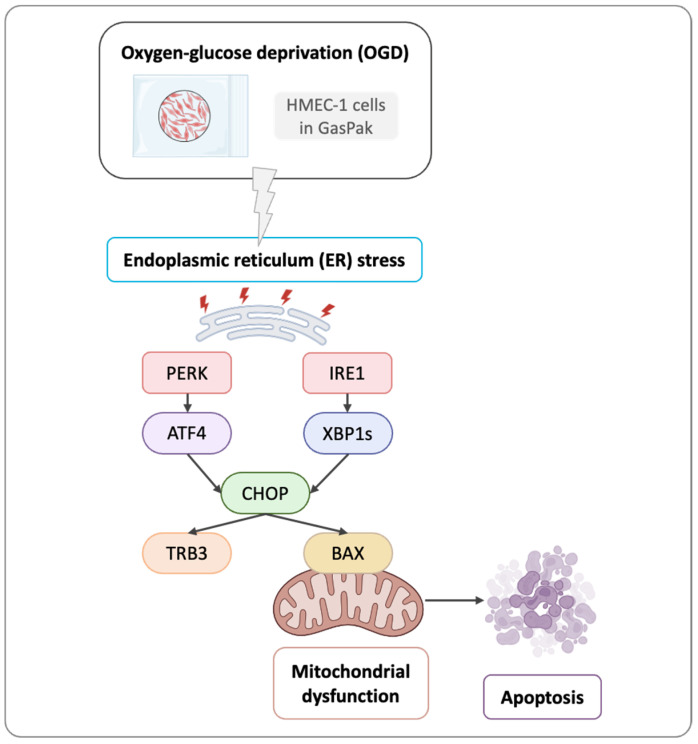
Graphical overview of the activation of two unfolded protein response pathways, PERK and IRE1. PERK and IRE1 lead to CHOP up-regulation, which induces apoptosis in HMEC-1 during OGD using GasPak EZ Pouch Systems.

All three genes (ATF4, XBP1s, and TRB3) were upregulated in HMEC-1 OGD-treated cells, when compared to controls. These findings suggest that OGD triggers UPR activation in HMEC-1 cells (Figure 6). Thus, OGD activates ER stress through IRE1 and PERK pathways, and presumably leads to CHOP-mediated pro-apoptotic pathways.

## 4. Discussion

During ischemic events, endothelial cells play a pivotal role in maintaining vascular homeostasis; therefore, preserving their structural and functional integrity may help improve outcomes after ischemic injury. Given its role as a core component of the vascular unit, the endothelium has naturally emerged as a promising therapeutic target in ischemia [18]. Furthermore, increasing evidence highlights the significant role of endoplasmic reticulum (ER) stress in ischemic processes where cells are subjected to severe metabolic stress which disrupts ER homeostasis and may lead to damage and cell death [1].

As metabolic pathways such as mitochondrial respiration and glycolysis are the primary sources of cellular ATP [19], and hypoxia is a key regulator of mitochondrial dysfunction [16], we studied the impact of OGD on HMEC-1 mitochondrial respiration. After OGD, glucose deprivation markedly reduced ATP production relative to control. Consistently, oxygen-consumption analyses showed reductions in basal respiration, ATP-linked respiration, proton leak, maximal respiration, spare respiratory capacity, and non-mitochondrial respiration. Together, these data suggest that OGD induces metabolic rewiring and pronounced mitochondrial dysfunction in HMEC-1, these cellular events limit energy production and may lead to cellular failure and death if sustained. OGD likely shifts metabolism toward anaerobic glycolysis at the expense of mitochondrial respiration. Since lactate production is an indirect readout of glycolytic flux, and given that lactate is the end product of anaerobic glycolysis, quantifying it after 8 h of OGD would strengthen this hypothesis [20].

Given these results and, as oxygen–glucose deprivation imposes severe metabolic stress, we evaluated cell viability following OGD in HMEC-1. The data indicated that cell death in OGD-induced cells may primarily follow the canonical programmed cell-death response to severe cellular damage and stress. Additionally, secondary necrosis was observed in OGD-treated cells. This increase parallels findings in oxygen–glucose deprivation and reperfusion (OGD/R)-treated human umbilical vein endothelial cells (HUVECs) [21], where elevated secondary necrosis has also been reported. Furthermore, BAX staining supported engagement of canonical mitochondrial apoptosis in OGD-treated HMEC-1 compared to normoxic controls. Further investigation via cleaved-caspase detection would strengthen interpretations about the apoptotic pathway. It should be noted that MTT assays should not be performed to assess cytotoxicity during OGD.

We studied ER stress regulation since prolonged ER stress can drive UPR-mediated cytotoxicity and apoptosis [2]. CHOP, a key effector of ER stress-induced apoptosis [3], was up-regulated in HMEC-1 cells following OGD compared to cells in normoxic conditions. This up-regulation was confirmed at the protein level by immuno-blotting, and the RNA level by qPCR. Immunofluorescence further showed nuclear CHOP in OGD-treated HMEC-1, whereas the signal was absent under normoxic conditions. Expression of unfolded protein response genes increased in OGD-treated HMEC-1 cells, including upstream effectors (ATF4, XBP1s) and a CHOP target (TRB3). Taken together, these findings indicate UPR activation in this model. A likely mechanism is that OGD elicits ER stress by activating the PERK-ATF4 and IRE-1-XBP1s arms of the UPR, ultimately leading to CHOP induction. CHOP then stimulates TRB3 and can promote pro-apoptotic signalling through BAX and subsequent apoptosis in HMEC-1 cells [22,23]. Overall, ER stress can drive HMEC-1 apoptosis via PERK and IRE1 pathway activation under OGD. In line with this interpretation, Badiola et al. reported PERK and IRE1 activation under OGD. However, despite ATF4 upregulation, CHOP did not increase in their model, highlighting context-dependent UPR outputs [24]. Conversely, with 16 h OGD in PC12 cells, hypoxia modulated the UPR and promoted autophagy instead of apoptosis, demonstrating that responses vary depending on cell model and OGD duration [25]. In any case, if this system is to be used in the future, investigators will need to demonstrate the causal relationship between CHOP activation and the apoptotic response in their specific study context, such as testing therapeutic molecules or researching the biological function of genes, functional RNA, or proteins.

It is commonly accepted that HMEC-1 retains key phenotypic and functional features of the microvascular endothelium and is widely used as an in vitro endothelial model [26]. In vitro, OGD provides a controllable approximation of ischemic conditions across tissues. A literature review shows no consensus on experimental parameters, including the composition of ischemia media and the duration of OGD. This lack of standardization in cell models and OGD conditions limits comparative analyses. Nevertheless, to more accurately replicate in vivo ischemic microenvironments, OGD can be conducted in co-cultures with relevant support cells. Such multicellular systems help investigate cell-survival pathways under ischemic stress [27]. This simple method describes here using GasPak can easily be upgraded for 3D culture.

## 5. Conclusions

In conclusion, this study examined the interplay between ER stress and ischemia using an oxygen–glucose deprivation protocol (OGD) in human microvascular endothelial cells-1 (HMEC-1). We evidenced the associated cell death and determined that, under OGD, mitochondrial respiration and ER homeostasis were disrupted, and two UPR pathways (PERK and IRE1) are activated. Activation of PERK and IRE1 pathways resulted in CHOP up-regulation and its nuclear relocalisation, which subsequently induced apoptosis in endothelial cells (Figure 6). *In fine*, these findings support the use of the GasPak EZ Pouch Systems to induce ischemic injury in vitro. It could also help to explore therapeutic options in various ischemic contexts, with the aim of restoring ER homeostasis and mitochondrial function, and ultimately limiting cell death. Taken together, the accessibility and robustness of this model position it as a valuable translational platform for the screening of ER stress modulators and the preclinical evaluation of therapeutic strategies targeting ischemia-related pathologies when hypoxia chambers or incubators are not available.

## Figures and Tables

**Table 1:** Current methods to induce hypoxia in cell culture.

Method	Principle	Advantages	Limitations	Typical O_2_ Level	References
Hypoxia chambers/controlled incubators	O_2_, CO_2_, and humidity precisely regulated using premixed gases	Physiologically relevant; stable and reproductible	Expensive equipment; slow transition between normoxia/hypoxia	0.1–5%	[5,6]
Chemical mimetics (CoCl_2_, DFO, DMOG)	Stabilize HIF-1α by inhibiting prolyl hydroxylases or chelating Fe^2+^	Simple; inexpensive; no special equipment needed	Off-target effects; do not reduce O_2_ directly	Normoxia, but “hypoxia-mimicked”	[7,8]
Oxygen scavenging systems	Enzymatic/chemical consumption of dissolved O_2_	Rapid induction; small-scale; low cost	Hard to maintain stable levels; alters pH/nutrients	Variable, often <1%	[9]
Microfluidic devices/organ-on-chip	Controlled O_2_ gradients via microchannels and oxygen-impermeable membranes	High spatially temporal resolution; mimics in vivo gradients	Technically demanding; limited throughput	Tunable (0–21%)	[10,11]
Physical diffusion barriers	O_2_ diffusion limited by extracellular matrix or media depth	Models tumors-like hypoxic gradients	Poor control of exact O_2_ level; variable reproducibility	Typically <5% in core regions	[12]

## Data Availability

Dataset available on request from the authors The raw data supporting the conclusions of this article will be made available by the authors on request.

## Supplementary Materials

The following supporting information can be downloaded at: https://www.mdpi.com/article/10.3390/mps9020039/s1, Figure S1: EF5 positive cells quantification during OGD treatment as in Figure 1; Figure S2: Flow cytometry analysis. The gating strategy consisted of sequential exclusion of debris, doublets, and non-viable events (FSC/SSC), followed by Annexin V and PI based discrimination of Q12, viable cells (Annexin V-, PI-), Q9, early apoptotic cells (Annexin V+, PI-), Q10, secondary necrotic cells (Annexin V+, PI+), and Q11, necrotic cells (Annexin V-, PI+).

## Abbreviations

ATF	activating transcription factor
BAX	Bcl-2–associated X
BBB	blood–brain barrier
CHOP	C/EBP homologous protein
ER	endoplasmic reticulum
GRP	glucose-regulated protein
HMEC-1	human microvascular endothelial cells-1
IRE1	inositol-requiring enzyme 1
MTT	3-(4,5-dimethylthiazol-2-yl)-2: 5-diphenyl tetrazolium bromide
OCR	oxygen consumption rate
OGD	oxygen–glucose deprivation
PERK	protein kinase RNA (PKR)-like ER kinase
PI	propidium iodide
TRB3	tribbles homolog 3
UPR	unfolded protein response
WB	Western blot
XPB1s	spliced X-box binding protein 1
