# Supplementary material for: A GasPak-Based Ischemia Model for Studying ER Stress–Ischemia Interactions in Human Endothelial Cells
Source: Methods Protoc. 2026 Mar 4;9(2):39. doi: 10.3390/mps9020039 (PMC13010650; doi:10.3390/mps9020039)
Supplement: Supplementary file 1 [file mps-09-00039-s001.zip › mps-4057731-supplementary.pdf]

Supplemental Information

A GasPak-based ischemia model for studying ER stress–ischemia interactions in human endothelial cells

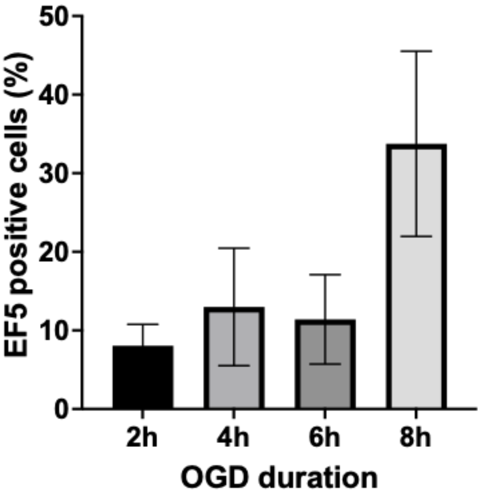

Figure S1. EF5 positive cells quantification during OGD treatment as in figure 1.

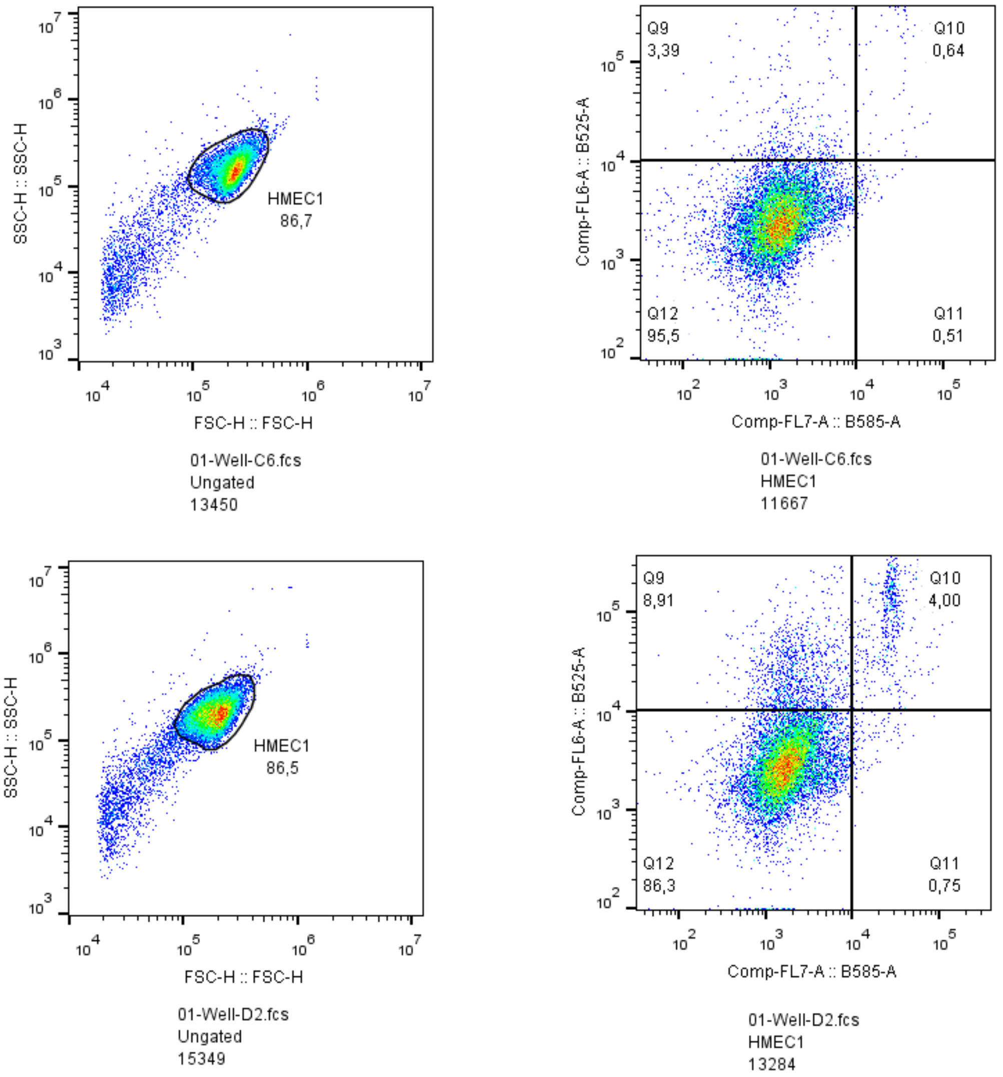

Figure S2. Flow cytometry analysis. The gating strategy consisted of sequential exclusion of debris, doublets, and non-viable events (FSC/SSC), followed by Annexin V and PI based discrimination of Q12, viable cells (Annexin V-, PI -), Q9, early apoptotic cells (Annexin V+, PI-), Q10, secondary necrotic cells (Annexin V+, PI+), and Q11, necrotic cells (Annexin V-, PI+).
